# Expression of progenitor markers is associated with the functionality of a bioartificial adrenal cortex

**DOI:** 10.1371/journal.pone.0194643

**Published:** 2018-03-29

**Authors:** Mariya Balyura, Evgeny Gelfgat, Charlotte Steenblock, Andreas Androutsellis-Theotokis, Gerard Ruiz-Babot, Leonardo Guasti, Martin Werdermann, Barbara Ludwig, Tobias Bornstein, Andrew V. Schally, Ana Brennand, Stefan R. Bornstein

**Affiliations:** 1 University Hospital Carl Gustav Carus, Dept. of Medicine III, Technische Universität Dresden, Dresden, Germany; 2 Centre for Endocrinology, William Harvey Research Institute, Queen Mary University of London, London, United Kingdom; 3 Paul Langerhans Institute Dresden of Helmholtz Centre Munich at University Clinic Carl Gustav Carus of TU Dresden Faculty of Medicine, Dresden, Germany; 4 Center for Regenerative Therapies, Technische Universität Dresden, Dresden, Germany; 5 Diabetes and Nutritional Sciences Division, King's College London, London, United Kingdom; 6 Divisions of Endocrinology and Hematology–Oncology, Departments of Medicine and Department of Pathology, University of Miami, Miller School of Medicine, Miami, FL, United States of America; 7 Veterans Affairs Medical Center, Miami, FL, United States of America; Cooper Medical School of Rowan University, UNITED STATES

## Abstract

Encapsulation of primary bovine adrenocortical cells in alginate is an efficacious model of a bioartificial adrenal cortex. Such a bioartificial adrenal cortex can be used for the restoration of lost adrenal function *in vivo* as well as for *in vitro* modeling of the adrenal microenvironment and for investigation of cell–cell interactions in the adrenals. The aim of this work was the optimization of a bioartificial adrenal cortex, that is the generation of a highly productive, self-regenerating, long-term functioning and immune tolerant bioartificial organ. To achieve this, it is necessary that adrenocortical stem and progenitor cells are present in the bioartificial gland, as these undifferentiated cells play important roles in the function of the mature gland. Here, we verified the presence of adrenocortical progenitors in cultures of bovine adrenocortical cells, studied the dynamics of their appearance and growth and determined the optimal time point for cell encapsulation. These procedures increased the functional life span and reduced the immunogenicity of the bioartificial adrenal cortex. This model allows the use of the luteinizing hormone-releasing hormone (LHRH) agonist triptorelin, the neuropeptide bombesin, and retinoic acid to alter cell number and the release of cortisol over long periods of time.

## Introduction

Adrenal insufficiency is a life-threatening disorder that requires a complex and permanent hormone replacement. Congenital adrenal hyperplasia (CAH) due to 21-hydroxylase deficiency is the most common form of adrenal insufficiency. Current treatment options with glucocorticoid substitution can only partially reverse the symptoms and exhibit unpleasant side effects [1]. Despite different treatment regimens, CAH remains a major therapeutic challenge sometimes requiring drastic therapeutic procedures such as bilateral adrenalectomy [2]. Transplantation of adrenal cells may be a feasible therapeutic alternative for those patients. However, this strategy is critically limited by a persistent lack of human donor organs and the requirement of chronic immune suppression.

Those problems could be solved by the transplantation of immunoisolated xenogeneic adrenal cells. Recently, we succeeded in creating a bioartificial adrenal cortex—a transplant, developed on the base of 3D xenogeneic primary Bovine Adrenocortical Cell (BAC) cultures, embedded in alginate [3]. This improved both the capacity of the adrenal cells for stable, long-term basal cortisol release as well as the response to stimulation with pituitary adrenocorticotropic hormone (ACTH). In addition, we successfully transplanted these bioartificial adrenal cortices intraperitoneally and subcutaneously into rats for a short period of time (approx. 3 weeks) within an implantable medical device without the use of immunosuppressive drugs [3].

Here, we improved this bioartificial adrenal cortex system by generating a long-term functioning, highly productive bioartificial organ that aims to induce minimal immunological responses. In this system, BACs were expanded *in vitro* in monolayer cultures for 1–7 days and then embedded in an alginate matrix for 3D growth for additional 114 days.

Because most BACs are mature cells with limited life span, it is essential that the cells in the bioartificial adrenal cortex include a population of adrenocortical progenitors. Previous reports showed the presence of adrenocortical progenitor cells in the adult rat adrenal cortex [4], suggesting that appropriate modeling of this organ should include immature cell populations. The presence of a side population of cells with stem and progenitor properties has also been demonstrated in mice [5, 6]. Experiments with transplantation of primary adrenocortical cells indirectly support the existence of adrenocortical progenitors in primary cultures of adrenocortical cells [5]. However, to our knowledge, no direct evidence of the presence of adrenocortical progenitor cells in primary cultures of bovine adrenocortical cells has been demonstrated.

Recently, the Sonic Hedgehog (Shh) signaling pathway was demonstrated to play an important role in the development of the adrenal cortex and to represent a population of undifferentiated cells [4, 7]. Shh-expressing cells are long-lived nonsteroidogenic progenitors of all steroidogenic cell types [8]. Additionally, these cells display a relatively high level of proliferation [4] and could replace apoptotic mature steroidogenic cells, thereby increasing the functional life span of the bioartificial adrenal cortex. Therefore, we investigated the expression of a transcription factor induced by Hh signaling, GLI1 and its receptor Patched1 in our BAC cultures.

DAX1 is critical for maintenance of adrenocortical progenitor cells, as previously reported by other labs [9]. Therefore we employed DAX1 as another marker of adrenocortical progenitors.

Nestin was originally identified as a marker of neural progenitor cells [10, 11]. However, later it was also shown to identify stem/progenitor cells in various tissues, including muscle, umbilical cord blood, testis, odontoblasts, hair follicle sheath, liver, and kidney [12]. Nestin-positive cells are also present in the human and murine adrenal cortex [13–15]. We have previously shown that Nestin-positive progenitor cells reside in the murine adult adrenal medulla and respond to stress by differentiation [16]. Another study revealed that Nestin-positive cells isolated from pancreatic islets are multipotent andable to differentiate into endocrine, exocrine and hepatic phenotypes [17]. In this research we reveal that Nestin-expressing cells originating from the adrenal cortex are sensitive to stimulation with ACTH. These observations suggest Nestin as an alternative progenitor marker.

Our concept is to identify adrenocortical stem and progenitor cells in the BAC culture, to determine the optimal time point for the onset of the 3D culture phase, when the cell culture has the maximal amount of progenitors, and to balance the volume of progenitors with the functionality of the bioartificial adrenal with the help of pharmacological agents. All these measures were designed to improve the outcome of adrenal replacement by transplantation of a bioartificial adrenal cortex.

## Materials and methods

### Cell preparation and culture

Adrenocortical cells were isolated from bovine adrenal glands shortly after slaughtering of 1–3 years old cattle as previously described [3]. Briefly, adrenal glands were transported to the laboratory in ice-cold Euro Collins Solution supplemented with 1% (vol/vol) penicillin-streptomycin solution (Thermo Fisher Scientific). The glands were then liberated from fat and connective tissue and rinsed several times with PBS through the central vein to remove remaining blood. Afterwards a longitudinal incision was made to cut the adrenals in halves, the medulla was removed and the cortex was scraped off the capsule and cut in small pieces. Adrenal capsule was discarded. Adrenal cortex was digested for 50 min in Dulbecco’s modified Eagle’s /Ham’s F12 (DMEM/F12) medium (Thermo Fisher Scientific), containing 2 mg/ml collagenase and 0.1 mg/ml DNase (both from Sigma-Aldrich) at 37°C while shaking. Obtained cells were washed with cultivation medium, pelleted by centrifugation (8 min, 300 x *g*) and filtered through 100-μm cell strainers (Becton Dickinson). After that, primary adrenocortical cells were placed in cell culture flasks (Thermo Fisher Scientific) and cultivated at 37°C in a humidified atmosphere (95% air, 5% CO_2_) in DMEM/F12 medium with 10% (vol/vol) FBS, 10% (vol/vol) horse serum (both from Thermo Fisher Scientific), 0.1 ng/ml recombinant FGF-2 (PromoCell GmbH) and 1% (vol/vol) penicillin-streptomycin solution. Medium change was performed every 2–3 days. Those cells that were cultivated for 7 days were stimulated with 3 ng/ml ACTH_1-24_ (Synacthen, Sigma-tau Arzneimittel GmbH) for 24 h on the 5-6^th^ day of cultivation.

### Cell encapsulation

Encapsulation of bovine adrenocortical cells in alginate was performed as previously described [3]. Briefly, the cells were dissociated from the culture flasks by trypsinization (TripLE, Thermo Fisher Scientific), pelleted by centrifugation and gently mixed with 3.5% (wt/vol) sterile alginate (UP-MVG, Novamatrix) dissolved in Custodiol-HTK solution (H.S. Pharma). Then 30 μl of the mixture of cells and alginate was placed on a glass plate and cross-linked with 70 mM strontium chloride containing 20 mM Hepes. Each bioartificial adrenal cortex contained 2 x 10^5^ cells. During the cultivation period, the bioartificial adrenal cortices were stimulated with 3 ng/ml ACTH once a week for 24 h. Medium change was performed every 2–3 days.

### Steroid release and measurement

To reveal how ACTH stimulation affects relative gene expressions, isolated BACs were seeded in 6 well plates. For stimulation of cortisol production one half of the cells was incubated in cultivation medium containing 3 ng/ml ACTH_1-24_ for 24 h on day 1, 7 and 10 following the isolation procedure. The remaining cells were cultivated in standard cell culture medium. RNA was collected from both groups of cells at the indicated time points, and the influence of ACTH stimulation on the expression of progenitor markers was measured on day 1, 7 and 10 after cell isolation.

Basal cortisol and aldosterone was measured in the supernatants of cell culture after 24 h of cultivation. For stimulation of cortisol production the cells were incubated in cultivation medium containing 3 ng/ml ACTH_1-24_ for 24 h. The concentration of cortisol or aldosterone in cell culture supernatants was detected by cortisol ELISA (IBL). Stimulation index was calculated by division of ACTH stimulated cortisol by basal cortisol.

### Reverse-transcription and real-time PCR

Total RNA from bovine adrenocortical cells was isolated using the RNeasy Mini or Micro kit (Qiagen) where appropriate. DNA was eliminated using gDNA eliminator columns during RNA preparation according to the manufacturer’s protocol. For reverse transcription, up to 1μg of total RNA was converted to first-strand cDNA using M-MLV reverse transcriptase, reaction buffer, RNase inhibitor, dNTP mix and oligo(dT) 15/random hexamer primer according to the manufacturer’s instructions (Promega). Primers were designed by Primer-BLAST–NCBI software to span at least one intron to prevent nonspecific amplification of DNA remnants.

Real-time PCR was performed using SYBR green (Qiagen) and a Roche Light Cycler 1.5 (Roche). To normalize data, the *RPS9* gene was used as an internal control gene. Evaluation of different housekeeping genes in our laboratory (*GAPDH*, *β-actin*, *TBP*) revealed that *RPS9* is the most stable gene in our system. Typical genes used as internal controls (*GAPDH* and *β-actin*) increased their expression in cultured cells in response to traumatization compared to freshly isolated cells. These data corresponds with previously published results [18].Primers used are presented in Table 1.

**Table 1:** List of primers and amplification conditions for RT-PCR.

Gene	Primer sequence	Annealing temperature, °C	Product size (base pairs)
RPS9	F: CGGAACAAACGTGAGGTCT	60–65	126
	R: CGCAACAGGGCATTACCTTC		
Patched1	F: GCTGCGAGCGAAGTTTCAAA	60	174
	R: ACTCGTCCACCAACTTCCAC		
GLI1	F: AGACTCCAGCTCTGGACCG	63	188
	R: GACCTGGCAGTCCTTCTGTC		
Nestin	F: CCCTGGAGCAGGAGAAACAA	60	128
	R: AGCCTCTAGGAGGGTCCTGT		
IL1β	F: CTGCAGCTGGAGGAAGTAGAC	65	277
	R: GCCAGTCCTCGGGGTTATTC		
IL6	F: ACGAAAGAGAGCTCCATCTGC	63	71
	R: AATGGAGTGAAGGCGCTTGT		
SF1	F: GCTACGCCGCTGGACTTC	60	388
	R: CACGTGTTGCTGGAGGTTTG		
CYP17A1	F: GGGGACATCTTCGGGGCTGG	60	497
	R: CTCTGCAGCAGCCGGGACAT		
DAX1	F: TACTCTTCAACCCGGACCTGC	60	190
	R: AACAGTTCAGCCAGGGTGTT		

F: forward, R: reverse

### Immunofluorescence staining and microscopy

#### FDA/PI staining

Dual fluorescein diacetate and propidium iodide (FDA/PI) staining was applied to define the viability of the BACs. Stained cells were examined under the microscope (Axioplan, Carl Zeiss). Analysis was performed using Axiovision Software (Carl Zeiss). Viability was defined by the quantification of live (green) and dead (red) cells on microphotographs. To quantify cell number in bioartificial adrenals, the cells from six micrographs for each condition were counted using ImageJ 1.44 software. All images have been treated equally.

#### CYP11B1 staining

BACs were washed with PBS and fixed in 4% PFA in PBS for 30 min. After additional washing with PBS the cells were incubated in blocking solution (3% BSA, 0.1% Triton X-100 in PBS for 30 min at room temperature). Then the cells were washed with PBS and incubated with the primary antibody (CYP11B1, Santa Cruz Biotechnology Inc) overnight at 4°C, washed with PBS and incubated for 1 h in secondary antibody (donkey anti-goat IgG-FITC, Santa Cruz Biotechnology Inc) (both antibodies were diluted 1:100).

### Experiments with triptorelin, bombesin and retinoic acid

The luteinizing hormone-releasing hormone (LHRH) agonist triptorelin (decapeptyl, [3]LHRH) was provided by one of us (AVS). Triptorelin was dissolved in DMSO and then diluted in culture medium to a concentration of 10μM. Cell culture medium, containing DMSO in a concentration 0.1% (vol/vol) was used as a control. Synthetic bombesin (Bachem) was dissolved in 0.9% NaCl and then diluted in culture medium to a concentration 0.5 μg/ml. Retinoic acid (Sigma-Aldrich) was dissolved in absolute ethanol (VWR International S.A.S) and then diluted in culture medium to a final concentration of 5 μM. Cell culture medium containing 5x10^-4^% (vol/vol) ethanol was used as a control.

Bioartificial adrenal cortices were cultivated with triptorelin, bombesin, retinoic acid and their respective controls for 6 days starting the day after cell encapsulation in alginate. During this period bioartificial adrenal cortices received freshly prepared compound-containing medium every day. After withdrawal of the compounds the bioartificial adrenal cortices were cultivated in standard medium for three months.

### Statistical analyses

Quantitative data is represented as mean ± SEM. Statistical significance was determined by a two-tailed Student’s t-test or one- or two-way Analysis of Variance (ANOVA) with the post hoc Bonferroni’s multiple comparison test where appropriate by using GraphPad Prism 5 software. A value of p≤0.05 was considered as significant in all tests.

## Results

### Characterization of BAC growth during the monolayer expansion phase

#### Adrenocortical stem/progenitor cells are present in the primary culture of BACs

To demonstrate the presence of adrenocortical progenitor cell markers and the Shh pathway components *GLI1* and *Patched1* we used established PCR and Real Time (RT)-PCR methods. Additionally, we also detected the expression of *DAX1* as well as expression of the neural and adrenomedullary progenitor marker *nestin* (S1 Fig) in the monolayer cell culture of BACs. Western blot confirmed the protein expression of the markers in BAC cell cultures (S2 Fig).

**Fig 1:**
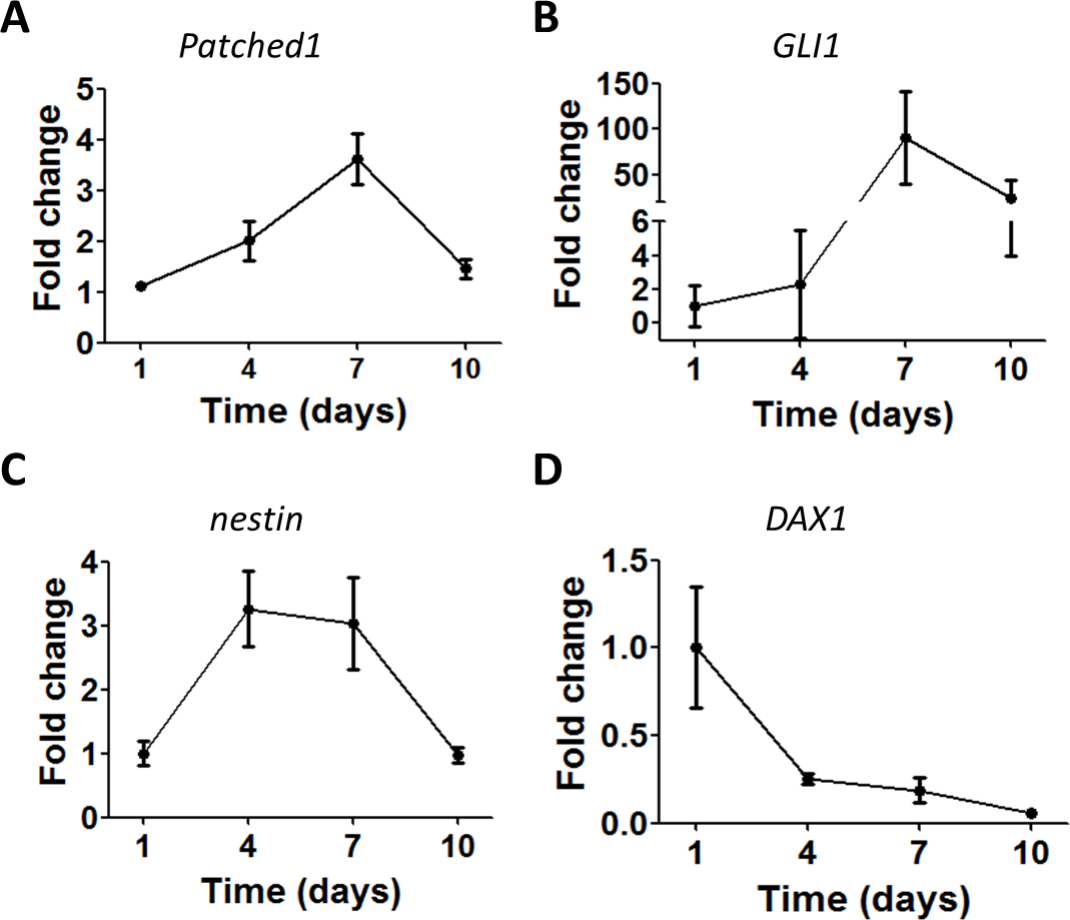
Dynamics of the relative gene expression of progenitor markers in primary cultures of bovine adrenocortical cells. RT-PCR analysis of the expression of *Patched1 (A)*, *GLI1 (B)*, *nestin (C) and DAX1 (D)* in in BAC cultures for a period of 10 days after cell isolation. All data presented as mean ± SEM, n≥3 for each sample. Reference gene *RPS9*.

We performed time-course experiments to select the time point with maximal gene expression of these markers. The results showed a progressive upregulation of gene expression for *Patched1* (2.3 fold, p = 0.06), *GLI1* (90.2 fold, p<0.05) and *nestin* (3 fold, p<0.01) from day 1 to 7 after cell isolation. Gene expression was downregulated from day 7 to 10 (Fig 1). *DAX1* followed a different course showing maximal expression at day 1.

#### Stimulation with ACTH increases gene expression of steroid hydroxylases and promotes differentiation of progenitor cells

Having established cultures with a relatively high level of progenitor biomarker expression, we next used pharmacological treatments to fine-tune the balance between progenitor markers and activity of mature BACs.

**Fig 2:**
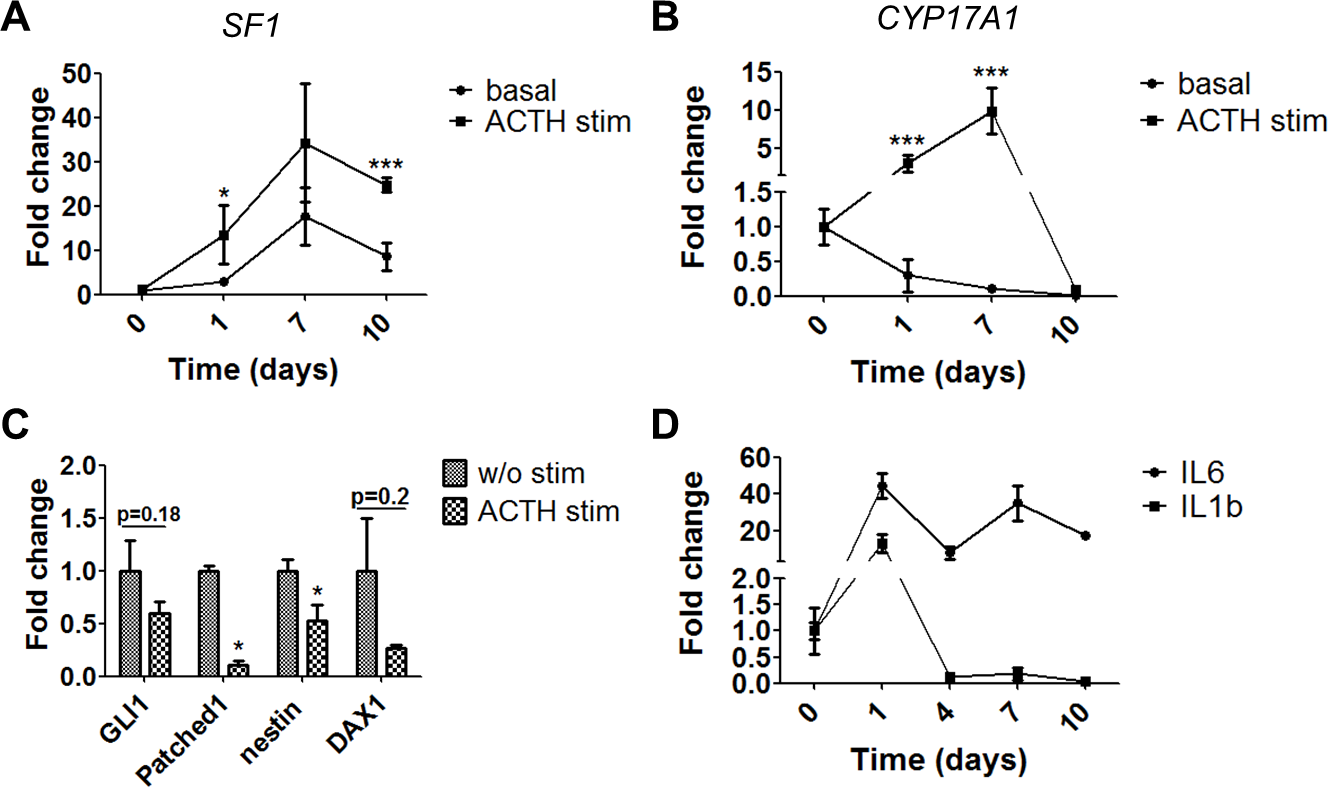
Dynamics of the relative mRNA gene expression of *SF1*, *CYP17A1* and interleukins 6 and 1β during cultivation of BAC. **A+B**–Dynamics of *SF1* (**A**) and *CYP17A1* (**B**) expression with and without ACTH-stimulation for a period of 10 days after cell isolation. **C**–Downregulation of progenitor markers after ACTH-stimulation on day 7 after cell isolation. **D**–Dynamics of the gene expression of *IL-6* and *IL-1β* 10 days after cell isolation. All data presented as mean ± SEM, n≥6 for each sample, *p≤0.05, ***p≤0.001. Reference gene *RPS9*.

ACTH helps to maintain the function of mature BACs. Specifically, ACTH stimulates the expression of cytochrome P450 (CYP) genes [19, 20], opposing the progressive reduction and eventual loss of steroid hydroxylases in BACs [21]. Therefore, we stimulated the BAC cultures with ACTH for 24 h. ACTH increased the expression of *SF1* (a proliferation marker of both progenitors and mature cells) and *CYP17A1* (a marker of mature cells) (Figs 2A and 2B). Specifically, the expression of *SF1* increased 4.8 fold for day 1 and 1.9 fold for day 7, and the expression of *CYP17A1* increased 10.5 fold for day 1 and 90.5 fold for day 7 compared to unstimulated cells. The influence of ACTH stimulation and cultivation length were analyzed by two-way ANOVA, and a significant effect of ACTH stimulation on both gene expressions was found (F = 6.86, p = 0.0124 for *SF1* and F = 15.77, p = 0.0003 for *CYP17A1*). The effect of cultivation length was also significant in both groups (F = 6.54, p = 0.001 for *SF1* and F = 7.3, p = 0.0005 for *CYP17A1* respectively). The stimulation has the same effect at all time points of cultivation for *CYP17A1* (F = 8.32, p = 0.0002). For *SF1* this effect was not significant (F = 0.83, p = 0.486).

ACTH treatment reduced the expression of the progenitor markers *Patched1* and *nestin*. A similar trend was observed with *GLI1* and *DAX1* but those data were not significant (Fig 2C). ACTH affects differentiation and steroidogenesis partly through SF1-induced transcription of transcription of steroidogenic enzymes [22], but DAX1, on the opposite, represses SF1-mediated transcription [23].

#### The dynamics of pro-inflammatory cytokines in BAC cultures

A bioartificial adrenal cortex should optimally not produce pro-inflammatory cytokines. We therefore investigated the expression of such cytokines in our BAC cultures. ELISA analysis of the supernatant did not detect the presence of IL-1β and TNFα (S1 Methods). RT-PCR analysis revealed that 24 h after cell isolation the expression of *IL-1β* was upregulated 12.9 fold and *IL-6* in 44.3 fold compared to freshly isolated cells. After 7 days in culture, the expression of these cytokines was reduced. The relative expression of *IL-1β* was 0.014 (p<0.01) and *IL-6* 0.78 (p>0.05 –not significant) on day 7 compared to day 1 after cell isolation (Fig 2D).

#### Optimal time point for the onset of the 3D bioartificial adrenal culture

**Fig 3:**
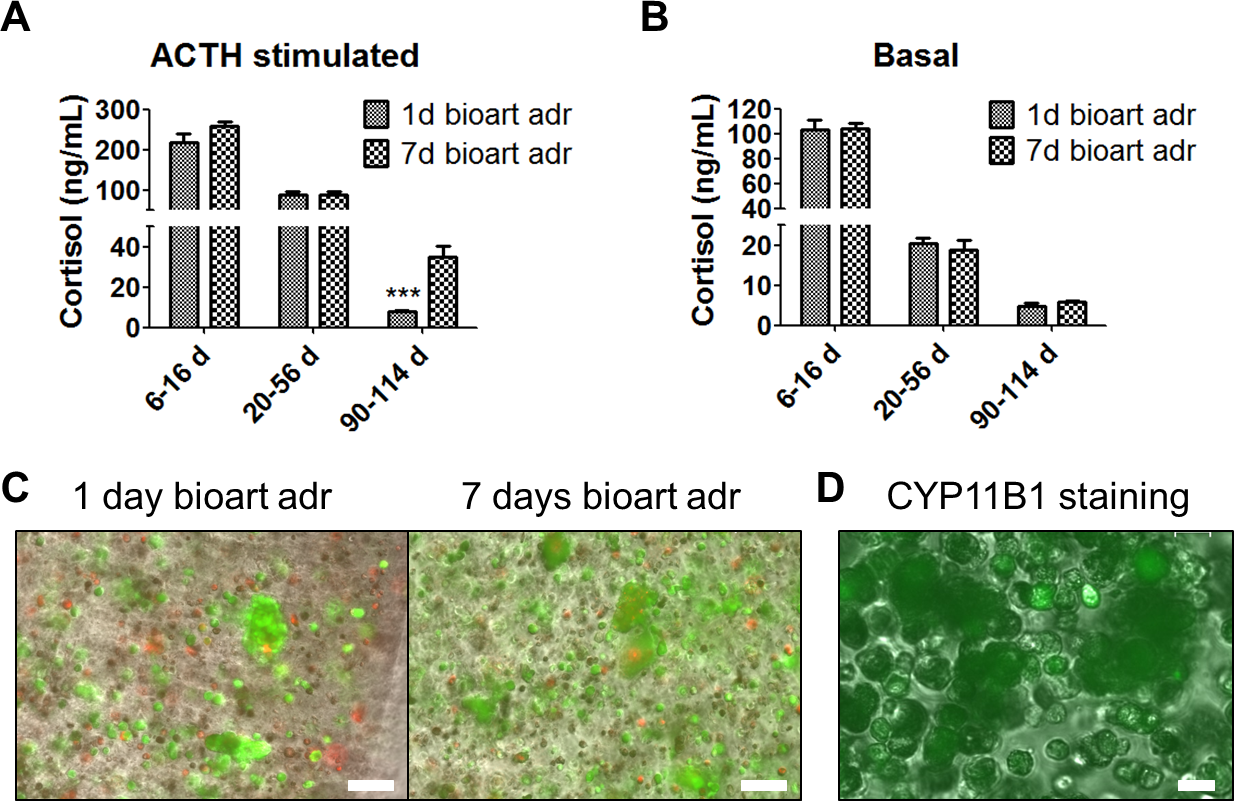
Comparative analysis of bioartificial adrenal cortices, created from BAC cultures, cultivated for 1 or 7 days after isolation before encapsulation in alginate. A–Dynamics of ACTH-stimulated cortisol production of bioartificial adrenal cortices during a cultivation period of 114 days (All data presented as mean ± SEM; n≥5 for each time point; ***p≤0.001). B—Dynamics of the basal cortisol release of bioartificial adrenal cortices during a cultivation period of 114 days (n≥5 for each time point). C–Cell cluster formation in bioartificial adrenal cortices formed from BACs 1 or 7 days after cell isolation (FDA/PI staining plus light microscopy). Scale bars = 100 μm. Uncropped images are seen in S4 Fig. D–Positive CYP11B1 staining (green) of cell clusters (Immunofluorescence plus light microscopy). Scale bar = 20 μm.

Given the differences in the BAC cultures between day 1 and 7 after cell isolation, we compared bioartificial adrenal cortices created at these two time points. Within the first two weeks after alginate encapsulation the average cortisol level was 103 ± 8 ng/ml for basal and 217±22 ng/ml for ACTH-stimulated day 1 bioartificial organs and 105±4 ng/ml and 258±10 ng/ml for day 7 bioartificial adrenal cortices, respectively. After 1–2 months of cultivation, both groups of bioartificial adrenal cortices produced similar amounts of cortisol (20.4±1.5 ng/ml for basal and 87.3±7.8 ng/ml for ACTH-stimulated day 1 and 18.7±2.4 ng/ml for basal and 89±7.7 ng/ml for ACTH-stimulated day 7 bioartificial adrenal cortices). After three months of cultivation, we detected a significantly higher ACTH-stimulated cortisol level in day 7 bioartificial adrenal cortices compared to day 1 (34.7±5.7 ng/ml vs. 8.1±0.4 ng/ml, respectively) (Fig 3A). Interestingly, we found no difference between basal cortisol productions in the two groups (Fig 3B). The stimulation index for day 7 bioartificial adrenal cortices was 5.9 and for day 1 it was 1.7. Basal aldosterone release of bioartificial adrenal cortices was determined two weeks after BAC encapsulation. Here, we found no difference in aldosterone production between the two groups (416±40 pg/ml for day 1 cortices and 391±37 pg/ml for day 7 cortices (p>0.1), respectively). The dynamics of aldosterone production was not evaluated. Moreover, microscopy of the bioartificial adrenal cortices revealed an increased cell cluster formation in day 7 bioartificial organs (Fig 3C and S4 Fig). The cell yield was assessed by the amount of cells. Cell counting at the end point of the experiment showed 3 times higher amount of cells (299±17.2% of day 1 bioartificial adrenal cortex, p<0.001) in bioartificial adrenal cortices that were created from 7 days old cell culture. CYP11B1 staining showed that cell clusters were formed from steroid producing cells (Fig 3D and S4 Fig), suggesting that the source for these cell clusters might be adrenocortical progenitor cells as well as transiently expressing cells.

Interestingly, we found that adrenocortical progenitor cells maintained their presence in alginate matrix. Seven day cell cultures expressed *GLI1* and bioartificial adrenals created from those cells also expressed *GLI1* at the end point of the experiment. On the contrary, 1 day old cell cultures (which did not express *GLI1* before encapsulation in alginate) failed to show *GLI1* at the end of the experiment (114 days in alginate). The viability of encapsulated cells in bioartificial adrenal cortices was 77±3% for day 1 and 87±1.3% for day 7, respectively (p<0.01).

### Altering the properties of the bioartificial adrenal cortex

We tested a range of pharmacological agents for their ability to influence the properties of the bioartificial adrenal cortex (S3 Fig). Many of these, including agonists of growth-hormone-releasing hormone (GHRH) (MR409, MR403, JI36 etc.) had no effect (S3 Fig). However, two compounds, triptorelin (LHRH agonist that in addition to stimulation of the secretion of luteinizing hormone and follicle-stimulating hormone, regulates the release of cortisol from adrenocortical cells [3, 24]), and bombesin (neuropeptide and growth factor that activates GLI1 and regulates proliferation in many progenitor cell types [25]) were shown to increase cell functionality and proliferative potential when applied on BACs encapsulated in alginate. Retinoic acid (metabolite of vitamin A that decreases basal cortisol in BACs [3] and promotes differentiation of progenitor cells [26]) in this improved bioartificial system also reduced the basal level of cortisol.

**Fig 4:**
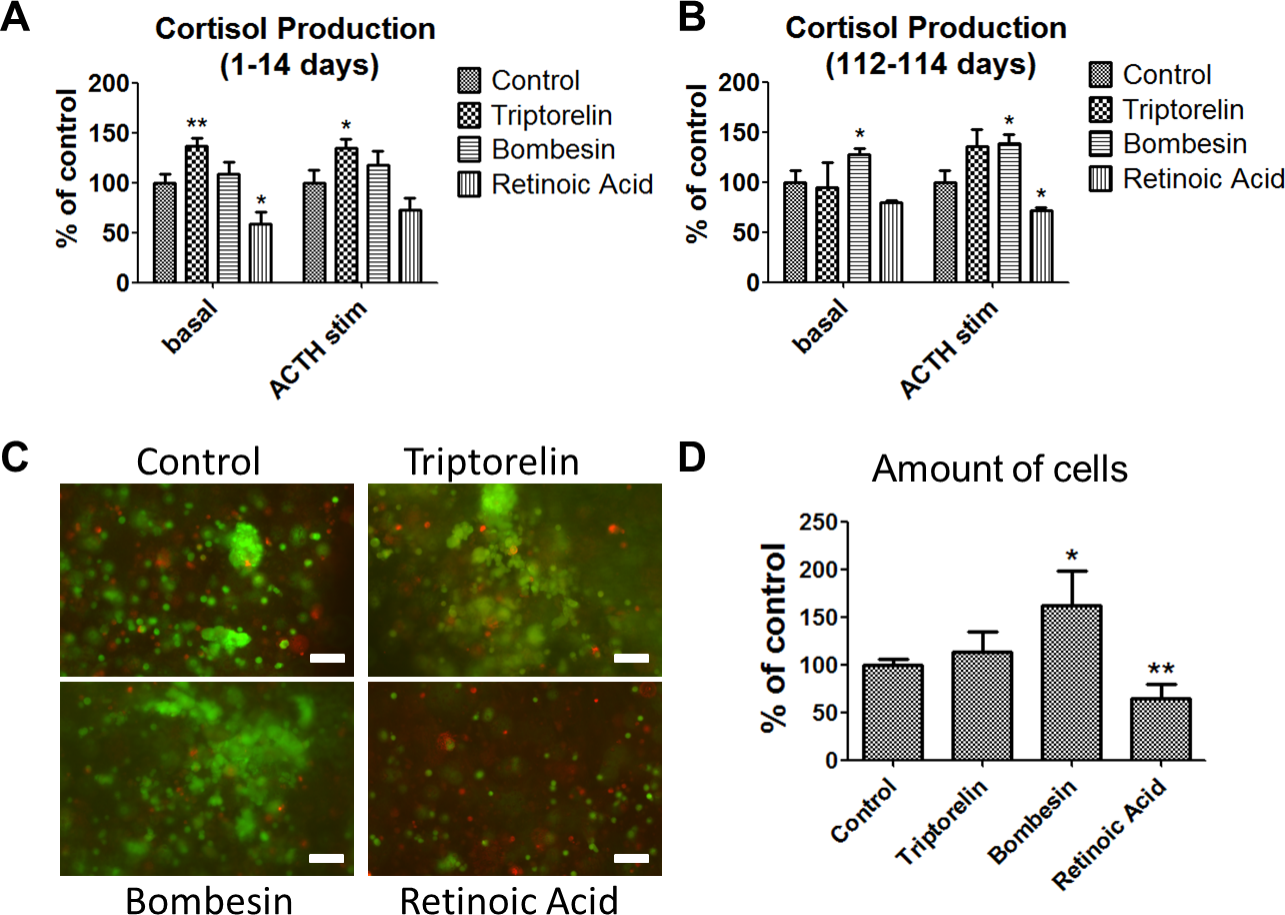
Short- and long-term effects of triptorelin, bombesin and retinoic acid on bioartificial adrenal cortices. **A**–Short-term effect of the compounds on both basal and ACTH-stimulated cortisol release within the first two weeks of treatment (all data presented as mean ± SEM, n≥18 for each group, *p≤0.05, **p≤0.01). **B**–Long-term effect of the compounds on basal and ACTH-stimulated cortisol levels at the end point of the experiment (112–114 days of cultivation). All data presented as mean ± SEM, n = 5 for each group, *p≤0.05. **C**–Cell cluster formation in different groups of bioartificial adrenal cortices after cultivation for three months (FDA/PI staining). Scale bars = 100 μm. Uncropped images are seen in S4 Fig. **D**—Amount of cells, counted in bioartificial adrenal cortices after cultivation for 114 days (n = 6 for each group).

More specifically, during the first two weeks of treatment with triptorelin, a significant increase in basal and ACTH-stimulated cortisol production was observed. In the triptorelin-treated group the basal cortisol reached 136.7±8.1% compared to control, and for ACTH-stimulated BACs it was 134.5±9.2%. Treatment with bombesin showed a tendency towards cortisol increase, which, however, did not reach significance. Retinoic acid reduced cortisol production (58.2±12.1% for basal, p<0.05, and 72.2±12.9% p>0.1 for ACTH-stimulated BACs) (Fig 4A).

We also tested for long-term effects in an experimental paradigm where the bioartificial adrenal cortices were treated for 6 days and then maintained untreated for more than three months. In this system the most pronounced effects were induced by bombesin. Here, bombesin increased basal (127.2±6.3% of control) and ACTH-stimulated (138.7±9% of control) cortisol levels. In contrast, retinoic acid showed a reduction of both basal and ACTH-stimulated cortisol to 79.4±18.4% of control, (p≥0.05) and to 71.2±11.3% of control, (p≤0.05), respectively). Cortisol production in the triptorelin-treated group showed a tendency to increase, but did not reach significance (Fig 4B). Moreover, bioartificial adrenal cortices treated with this compound, showed a different morphology of cell cluster formation (Fig 4C and S4 Fig).

The amount of cells was 113±22% of control (p>0.1) for the triptorelin-treated group, 162±36% (p<0.05) of control for bombesin and 64±16% (p<0.01) of control for bioartificial adrenal cortices treated with retinoic acid (Fig 4D). RT-PCR performed at the end point of the experiment showed no effects of the compounds on the expression of *SF1*,*CYP17A1* and the progenitor markers *Patched1*, *GLI1*, *DAX1* and *nestin* compared to their controls.

## Discussion

Here, we present an improved *in vitro* system that acts as a bioartificial adrenal cortex, based on cultures of BACs in an alginate matrix. Previously, we have shown that the application of alginate extracellular matrix on primary BAC cultures mimics several properties of the adrenal cortex [3]. Here, to generate an improved model, we optimized the conditions to maintain a progenitor cell population in the bioartificial adrenal cortex. This may lead to eventual application of this model in xenogeneic transplantation for the restoration of adrenal function in patients suffering from adrenal insufficiency or adrenal hyperplasia due to 21-hydroxylase deficiency and Addison’s disease.

Adrenocortical progenitor cells in cell culture might derive from the subcapsular region [27] during cell isolation. Our approach in modeling the composition of the adrenal cortex that contains both mature and progenitor cells was to isolate BACs, expand them in culture until the point (7 days), where the cultures contain the highest levels of progenitor markers, and then to use pharmacological treatments during the alginate culture phase to balance the content of progenitor biomarkers with the function of mature cells.

While working with primary cultures of adrenocortical cells, we have to take into consideration a progressive loss of steroid hydroxylases [21]. Stimulation with ACTH can significantly postpone this process [19, 20]. However, ACTH stimulation seems to downregulate the expression of adrenocortical progenitor markers, promoting differentiation of progenitor cells [28], which our data corroborate. Therefore, on one hand to prevent the loss of steroidogenic enzymes, adrenocortical cells need to be stimulated with ACTH. However, on the other hand, ACTH stimulation reduces the expression of adrenocortical stem/progenitor biomarkers in the culture, which in the long run might shorten the functional life-span of the created bioartificial organ. Precise refinements of this balance using pharmacological treatments may further improve this aspect of the method.

Production of pro-inflammatory cytokines by the graft is a limiting factor in transplantation approaches. Activation of cytokine signaling networks with a high production of proinflammatory interleukins is a universal response to any tissue damage occurring during primary cell isolation. Transplanted organs releasing cytokines would definitely cause a local host immune response. Cytokines could be produced not only by fibroblastic and macrophage elements, but also by the BACs themselves [29, 30]. However, as it has been already shown, proliferation and steroidogenesis of adrenocortical cells were closely linked to the level of IL-1β in BAC culture. Blocking the production of IL-1β significantly reduced or even abolished the elevation of proliferative activity and cortisol production by adrenocortical cells [31]. Similar effects were obtained in other studies. [32]. IL-6 also has a pronounced ability to stimulate proliferation and steroidogenesis [32–34]. Previous studies have demonstrated that inflammatory cytokines promote retrodifferentiation of mature cells to progenitor cells [35]. Overall, cytokines do not only play a pathogenic role, but also have an important physiological function in the activation of proliferative processes and steroidogenesis in adrenocortical cell cultures. In this study we show that, especially when cells that have been cultured for 7 days are selected to establish the bioartificial adrenal cortex, the levels of inflammatory cytokines are low.

Beyond differences in inflammatory cytokine production, the present study shows that BACs on day 1 and day 7 exhibit different biomarker expression profiles. The expression of *CYP17A1* and *DAX1* was highest on day 1 after cell isolation, whereas the expression of *GLI1*, *Patched1*, *nestin* and *SF1* reached their maximum on day 7. The next step was to compare bioartificial adrenal cortices generated from BACs on day 1 and 7 after the isolation procedure. During the cultivation period we did not find any significant difference in cortisol production between the two groups of bioartificial organs, but at later time points, we noticed the formation of cell clusters in the bioartificial adrenal cortices created from 7 days old cell cultures but considerably less from the 1 day old cell cultures. By this time we detected significantly higher ACTH-stimulated cortisol release in this group. The productivity of bioartificial adrenal cortices correlated with the amount of newly formed cell clusters. Cell count at the end point of the experiment confirmed an increased cell yield in the day 7 bioartificial adrenal cortices. Moreover, 7 days bioartificial adrenal cortices showed significantly higher viability at the end point of the experiment. The elevated amount of viable cells might be responsible for the higher ACTH stimulated cortisol production in this group, compared to 1 day bioartificial adrenal cortices.

Another gene that was highly expressed in 7 days old cell culture was *SF1*. The role of SF1 in the homeostatic proliferation of the adult gland has already been demonstrated [9]. Additionally, SF1 acts as the obligate activator of most steroidogenic enzymes in the adrenal cortex and plays an essential part in both proliferation and differentiation (steroidogenesis) of the adult gland [9]. It is important to note that BACs, cultivated for 7 days before encapsulation in alginate and showing maximal gene expression of *Patched1*, *GLI1 and nestin*, exhibited a better outcome in ACTH stimulated cortisol production when cultured for more than 90 days.

Even though encapsulation of 7 days old cell cultures in the long run significantly improved the functionality of bioartifical adrenal cortices, still long-term cultivation unfortunately led to a major decrease in cortisol levels. One of the reasons for this loss could be that bioartifical adrenal cortices were mostly generated from mature adrenocortical cells with limited life span. Finding a way to additionally enrich the source cell culture with adrenocortical stem and progenitor cells could solve this problem.

The bioartificial adrenal cortices are amenable to pharmacological manipulation. Therefore, we tested a range of pharmacological agents for their ability to influence the properties of the bioartificial adrenal cortex. These agents were relevant for adrenal function [24, 36–38], and furthermore, they were known to have the ability to stimulate self-renewal and promote survival of for example cardiac stem cells [39]. In addition, we have previously shown that some of the GHRH agonists can stimulate growth, function, and engraftment of pancreatic islets after transplantation [40, 41]. During the cultivation of bioartificial adrenal cortices in triptorelin-containing medium and 8 days after withdrawal of triptorelin, the LHRH agonist treated group showed an increased basal and ACTH-stimulated cortisol production. These data corroborate previously published results, showing a surge of steroid hormone release after administration of triptorelin [42]. Other studies demonstrated an effect of bombesin on steroid production [43], which we were not able to confirm. On the other hand, the bombesin results support its proliferative potential [25] as assessed by the amount of cells at the end point of the experiment. Retinoic acid produced both short-term (2 weeks) and long-term (~3–4 months) effects. Short-term effects were manifested as a significant reduction of cortisol production. Long-term effects were assessed at the later stages of the experiment, where we detected a reduced cell yield and ACTH-stimulated cortisol release.

Our work extends our studies and improves our previous efforts to develop bioartificial adrenal cortices with long-term survival capacity providing the ability to respond to pharmacological treatments. Such efforts may form the basis for eventual transplantation strategies for patients with severe adrenal disorders.

## Supporting information

S1 Methods Supplementary methods for S1–S4 Figs. (DOCX)

S1 Fig Reverse transcription PCR analysis of the expression of bovine *GLI1*, *RPS9*, *DAX1*, *nestin* and *Patched1* in isolated BACs. (TIF)

S2 Fig The protein expression profile in cells isolated from 4 different bovine glands cultured for 1, 4 and 7 days was examined by immunoblotting. Glyceraldehyde 3-phosphate dehydrogenase (GAPDH) was used as loading control. The samples were loaded from the left to the right as following: adrenocortical cells from adrenals 1, 2, 3 and 4 cultivated for 1 day after cell isolation, for 4 days and then for 7 days.
(TIF)

S3 Fig Effect of pharmacological agents on BACs during the monolayer expansion phase. All data presented as mean ± SEM, n≥3 for each sample, *p≤0.05, **p≤0.01.
(TIF)

S4 Fig Uncropped images from Figs 3C, 3D and 4C. (TIF)
