# Supplementary material for: Expression of progenitor markers is associated with the functionality of a bioartificial adrenal cortex
Source: PLoS One. 2018 Mar 29;13(3):e0194643. doi: 10.1371/journal.pone.0194643 (PMC5875767; doi:10.1371/journal.pone.0194643)
Supplement: S1 Methods — (DOCX) [file pone.0194643.s001.docx]

**S1 Methods**

**PCR**

For PCR analysis 370 ng of cDNA was amplified in a 10-μl solution of 1.5 mM MgCl_2_, 1× PCR buffer (Invitrogen), 0.25 mM of deoxynucleotides (Promega), 1 unit of Taq DNA Polymerase (Invitek), and 0.5 μM of forward and reverse primers. Samples were heated for 5 min at 94°C and then subjected to 15 s at 94°C, 15 s at 60°C (for *RPS9*, *nestin* *Patched1* and *DAX1*) or 63°C (for *GLI1*) and 15 s at 72°C for 40 cycles and finally amplified at 72°C for 1 min. Water was used as negative control.

**Western Blot**

Bovine adrenocortical cells were lysed in M-tissue cell lytic reagent (Sigma-Aldrich), and total protein content was determined using the Bradford protein assay (Sigma-Aldrich). 50 µg of the protein for each sample was separated on 10% SDS/PAGE gels and then transferred onto nitrocellulose membranes. After blocking in 5% nonfat dry milk for GLI1 or 5% BSA (both from Sigma-Aldrich) for other antibodies, these membranes were incubated overnight at 4°C with antibodies specific for DAX1/NR0B1, GLI1 (both from Antibodies-online GmbH), PTCH (St John’s Laboratory), Nestin (OriGene Technologies Inc.) or GAPDH (Cell Signaling Technology). After washing, the membranes were incubated for 1 h with goat anti-rabbit HRP conjugate (Bio Rad) for GAPDH, Nestin and GLI1, or donkey anti-goat IgG-HRP (Santa Cruz) for PTCH and DAX1/NR0B1. Signals were measured using SuperSignal West Femto substrate (Pierce, Thermo Scientific).

**Detection of IL-1β and TNF-α in cell culture supernatants**

After isolation, the cells were seeded in 6 well plates with 5x10^5^ cells per well in standard culture medium. After different lengths of cultivation (1, 2 or 3 days) the cell culture supernatants were collected. IL-1β and TNF-α in the cell culture supernatants were measured using the bovine IL-1 beta ELISA Reagent Kit (Thermo Fisher Scientific) and Bovine TNF-alpha ELISA (Raybiotech). In all cases the levels of both interleukins were below the minimal value of the calibration curve (31 pg/mL for IL-1β and 123 pg/mL for TNF-α respectively). Additional measurements with similar results were implemented using cell culture supernatants from cells cultivated in flasks up till 100% confluency.

**Experiments with GHRH agonists**

GHRH agonists MR409, MR502, MR356, MR367, MR403 and JI36 were dissolved in DMSO and diluted in cell culture medium to a final concentration 10 µM (IC_50_ for these analogs is 0.74-2.16 nM [44]. Cell culture medium, containing 0.1% (vol/vol) DMSO was used as a control.

The cells were treated with the pharmacological agents for 72 h. During this time the cells received freshly prepared medium every day.

**Assessment of proliferation, apoptosis and viability**

The day after the isolation BACs were seeded in 96 well plates with 1x10^4^ cells per well in four wells for each group. The cells were incubated with standard cell culture medium with or without pharmacological compounds. Proliferation was measured using Cell Proliferation ELISA BrdU (Roche) following the manufacturer’s protocol. Apoptosis was assayed by determination of caspase 3/7 activity using Caspase-Glo 3/7 Assay (Promega) according to the manufacturer’s instructions. Viability of BAC was defined by CellTiter 96® AQueous One Solution Cell Proliferation Assay (Promega) following the manufacturer’s protocol.

**Steroid release and measurement**

The day after isolation the cells were seeded in 24 well plates with 5x10^4^ cells per well in six wells for each condition. The cells were cultivated with or without pharmacological compounds in the cultivation medium. After 48 h of cultivation, three wells of each group of cells received stimulation medium, containing 3 ng/ml ACTH1-24 (Synacthen, Sigma-tau Arzneimittel GmbH) and other three – standard media (basal) with or without pharmacological agents.

The concentration of cortisol in cell culture supernatants was detected by ELISA (IBL). Stimulation index was calculated by division of ACTH stimulated cortisol by basal cortisol.
