# Supplementary figures and images for: Expression of progenitor markers is associated with the functionality of a bioartificial adrenal cortex
Source: PLoS One. 2018 Mar 29;13(3):e0194643. doi: 10.1371/journal.pone.0194643 (PMC5875767; doi:10.1371/journal.pone.0194643)

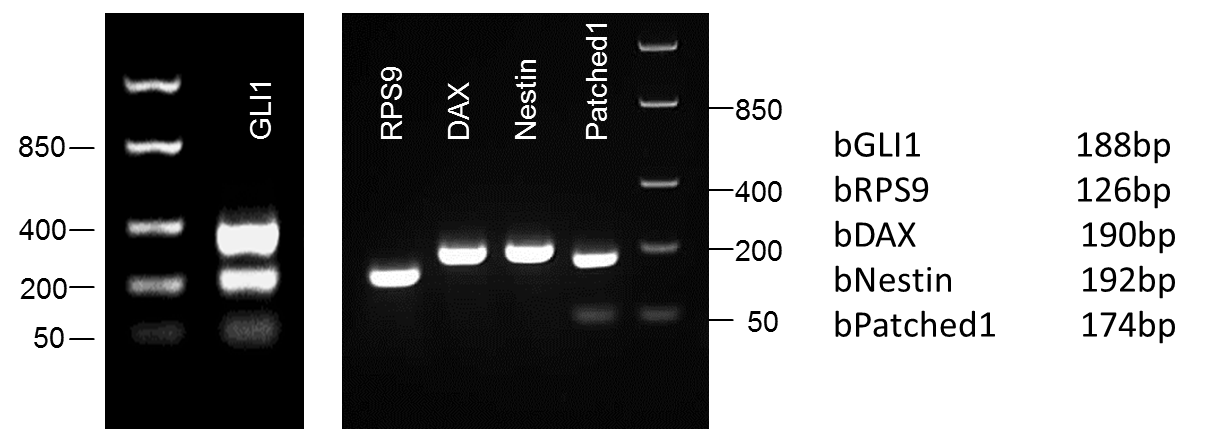

Supplement: S1 Fig — (TIF) [file pone.0194643.s002.tif]

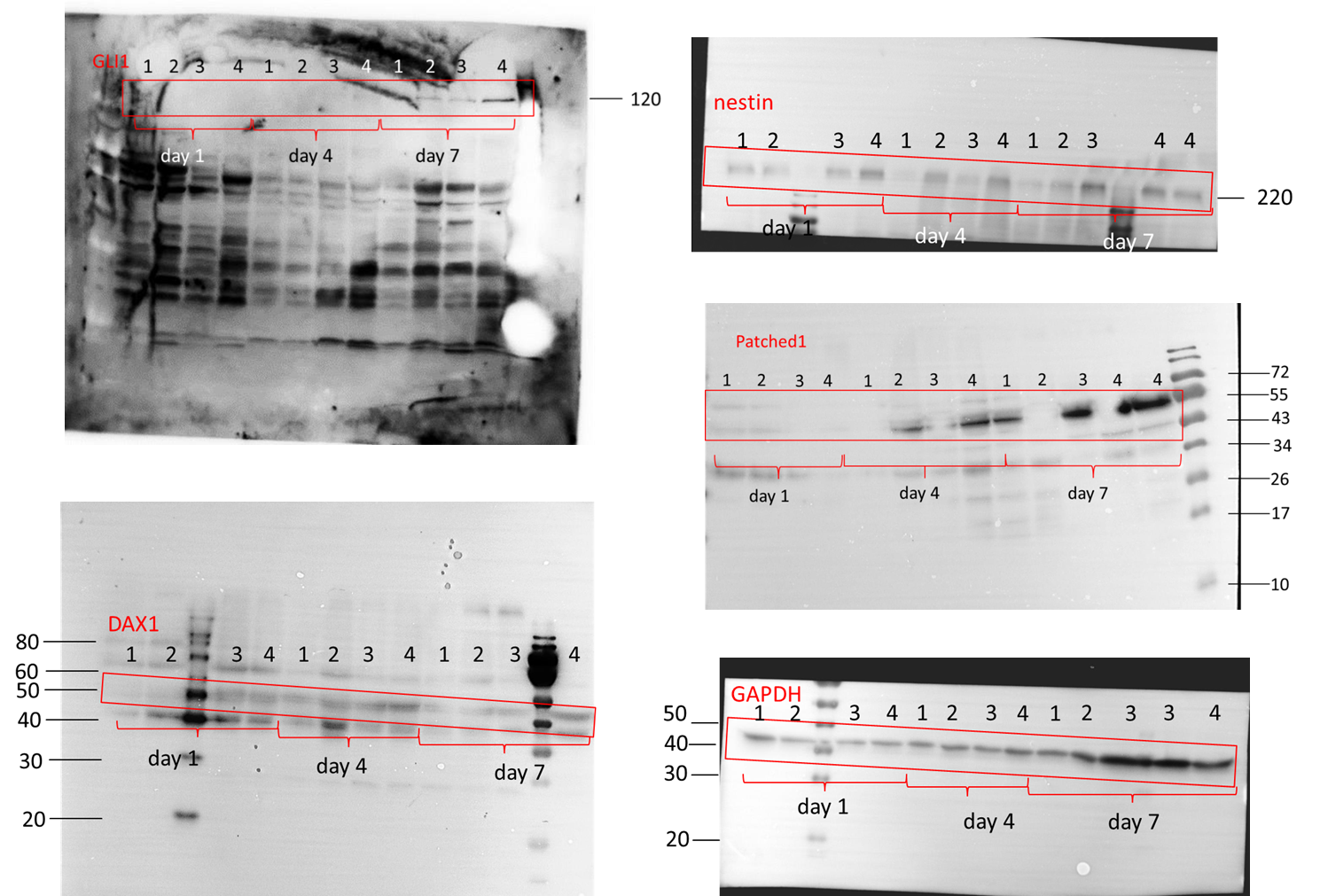

Supplement: S2 Fig — The samples were loaded from the left to the right as following: adrenocortical cells from adrenals 1, 2, 3 and 4 cultivated for 1 day after cell isolation, for 4 days and then for 7 days. (TIF) [file pone.0194643.s003.tif]

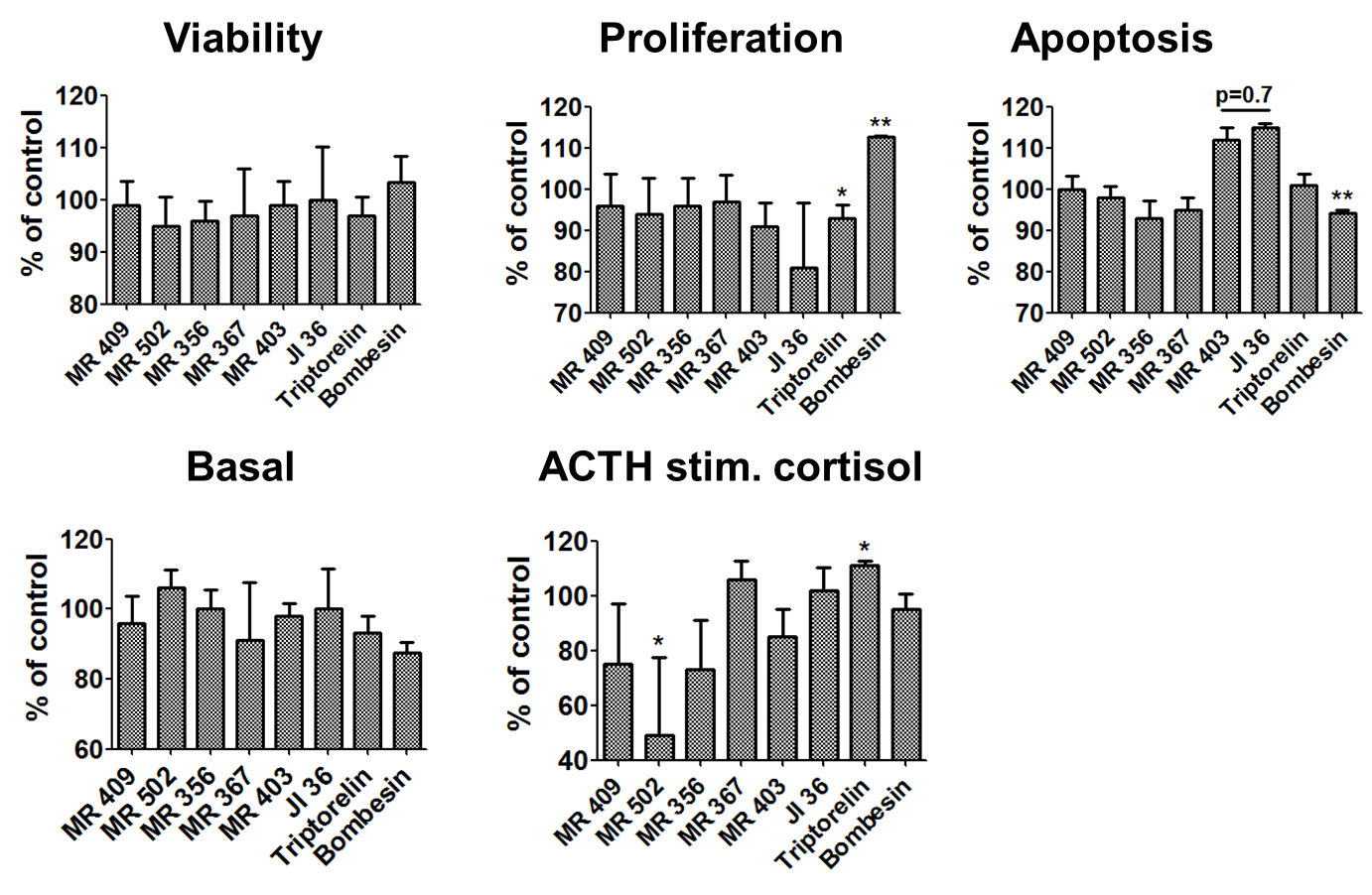

Supplement: S3 Fig — All data presented as mean ± SEM, n≥3 for each sample, *p≤0.05, **p≤0.01. (TIF) [file pone.0194643.s004.tif]

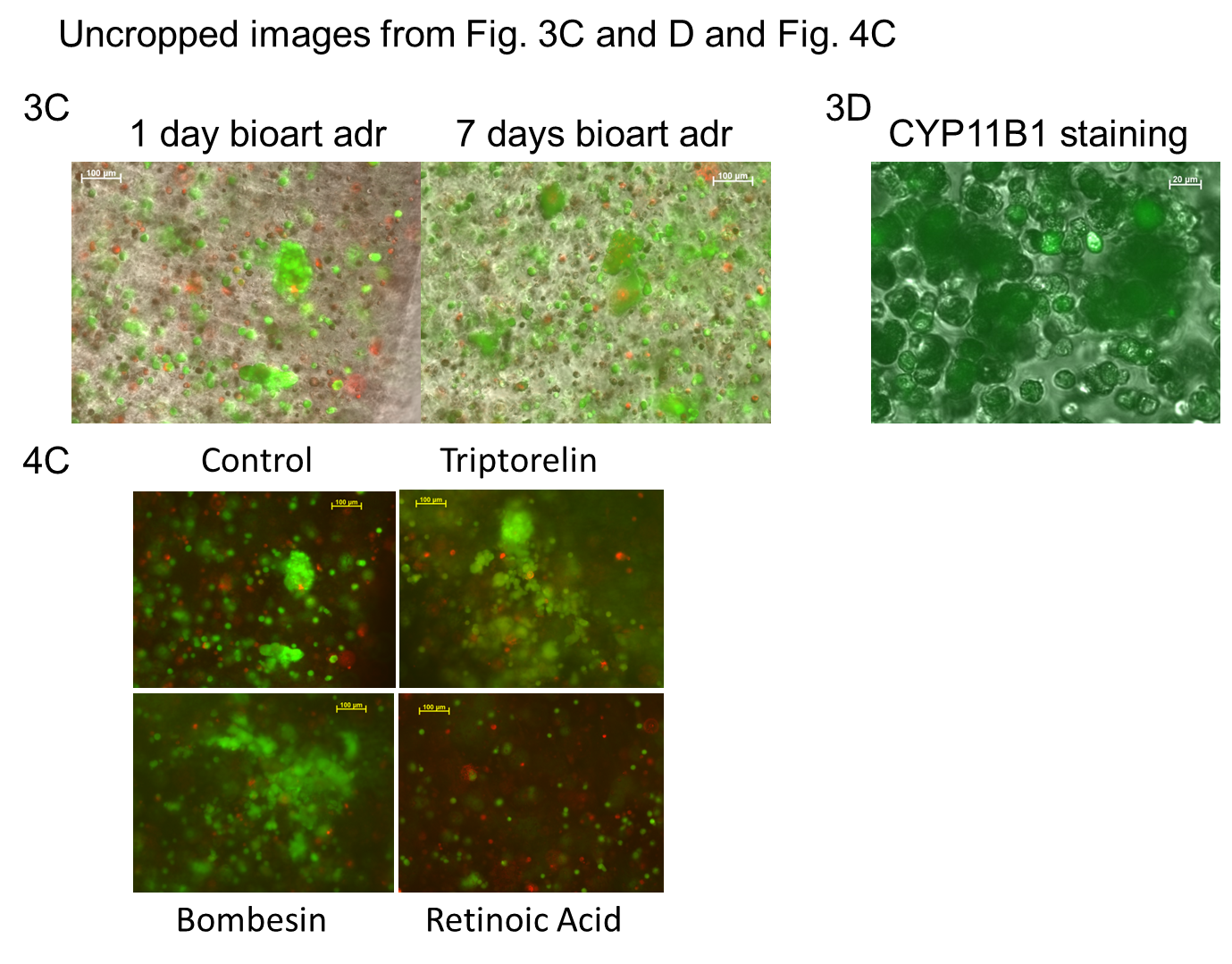

Supplement: S4 Fig — (TIF) [file pone.0194643.s005.tif]
